# Supplementary material for: Electronic metal-support interaction enhanced oxygen reduction activity and stability of boron carbide supported platinum
Source: Nat Commun. 2017 Jun 22;8:15802. doi: 10.1038/ncomms15802 (PMC5489685; doi:10.1038/ncomms15802)
Supplement: Supplementary Information — Supplementary Figures, Supplementary Tables, Supplementary Notes and Supplementary References [file ncomms15802-s1.pdf]

Supplementary Note 1. Determination of target Pt loadings on BC

We aim to keep the mass Platinum per surface area support constant across supports:

$$\frac{m_{Pt}^{C}}{A_C} = \frac{m_{Pt}^{BC}}{A_{BC}} \tag{S1}$$

The weight percent Platinum is defined as

$$x_C = \frac{m_{Pt}^{C}}{m_{Pt}^{C}+m_C} \text{ and } x_{BC} = \frac{m_{Pt}^{BC}}{m_{Pt}^{BC}+m_{BC}} \tag{S2}$$

Combining (S1) and (S2), the target weight percent Platinum on BC that yields the same mass Platinum per surface area as on C can be calculated from

$$x_{BC} = \frac{\gamma\left(x_C^{-1}-1\right)^{-1}}{\gamma\left(x_C^{-1}-1\right)^{-1}+1} \tag{S3}$$

with $\gamma = a_{BC}/a_C$ being the ratio of specific surface areas of both support materials. We used a value of $\gamma = 80.4/259 = 0.31$ to calculate the target weight percent on BC from the actual weight percent on C in Table S1.

Supplementary Note 2. Conductivity

The conductivity of the powders was measured using a custom-made conductivity device, the graphite rich boron carbide and Vulcan XC 72R conductivities were measured to be 3.8 ± 0.5 S/cm and 3.9 ± 0.5 S/cm at room temperature.

Supplementary Note 3. C 1s chemical shifts in B_4C from Density Functional Theory

Density Functional Theory (DFT) in the Generalised Gradient Approximation (GGA) as parameterised by Perdew, Burke, and Ernzerhof (PBE) was used to quantify the chemical shift of the C 1s core electrons within B_4C. The Partially Augmented Wave (PAW) formalism was used as implemented in Quantum Espresso V5.4.
The $B_{11}C_p$-CBC polymorph with experimental lattice parameters and atomic coordinates obtained from the Inorganic Crystal Structure Database (ICSD) were used (rhombohedral primitive cell with a=4.9424 Å and α=66.07°).
The C 1s core binding shift was calculated as the change in total energy between placing a C 1s core hole at C in tetrahedral coordination (CBC) and the polar position ($B_{11}C$), respectively:

$$\Delta = E^{1s^*}_{B_{11}C} - E^{1s^*}_{CBC}$$

A supercell approach was used to minimized spurious screened hole interactions between supercell images.

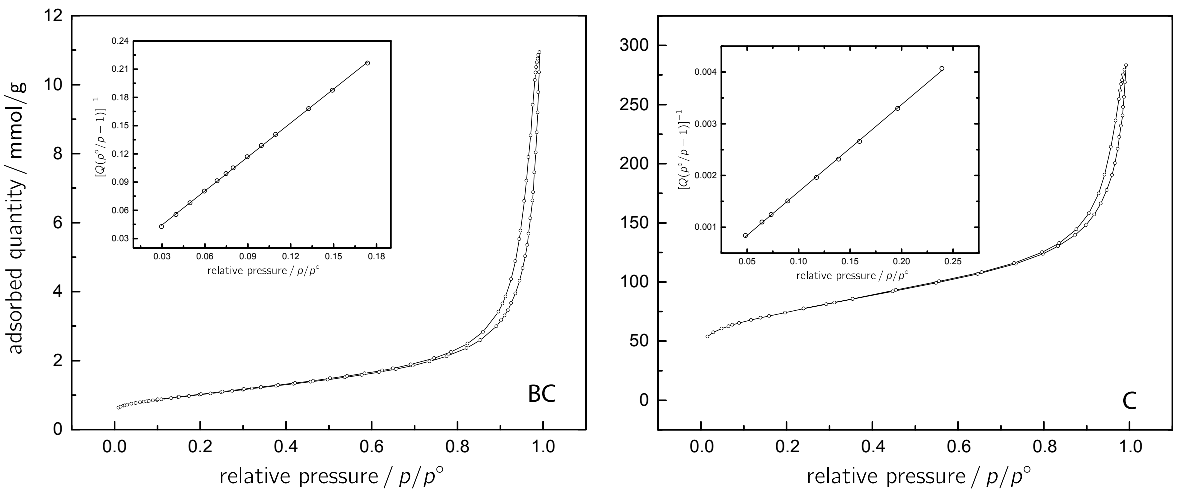

Supplementary Figure 1. Nitrogen adsorption isotherms for BC and C supports; insets show linear fit to the BET equation from which BET surface areas are obtained.

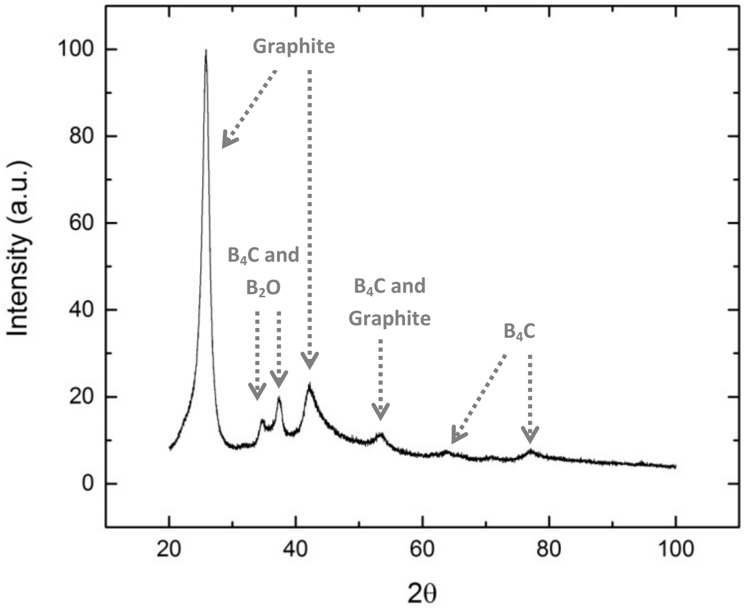

Supplementary Figure 2. X-ray diffraction pattern of the graphite rich boron carbide catalyst support

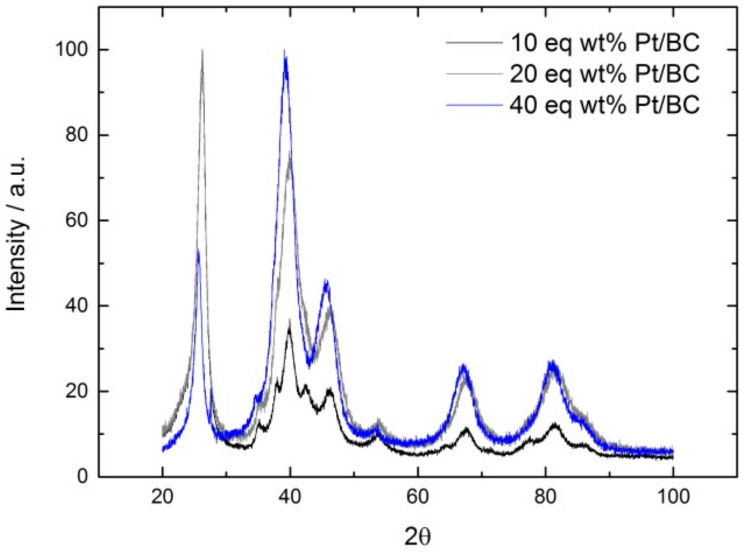

Supplementary Figure 3. X-ray diffraction pattern of the 10, 20 and 40 eq wt% Pt/BC catalysts

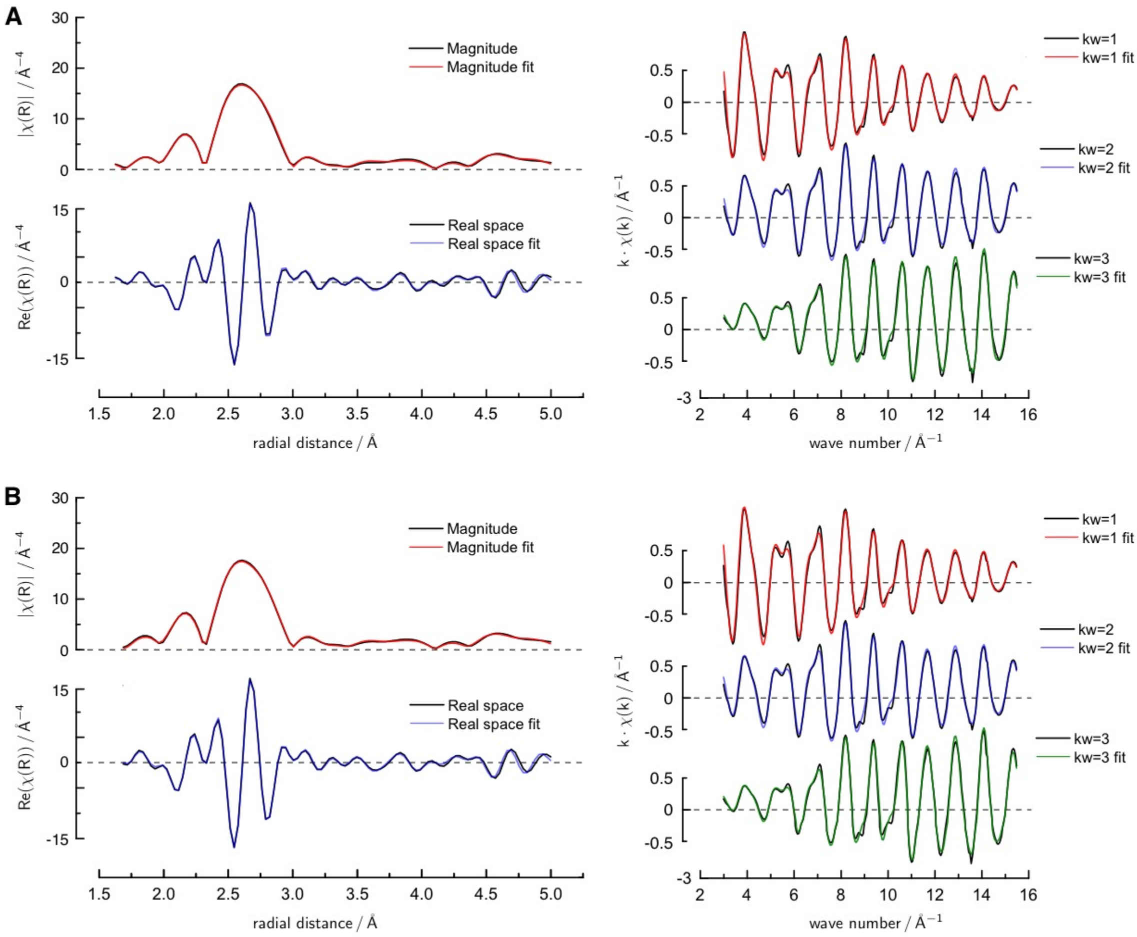

Supplementary Figure 4. Actual and modelled EXAFS data. A) R space and k space plots for the 20 eq wt% Pt/BC catalyst, k weighting (kw)=1 is scaled by 7.665, kw=2 is unscaled and kw=3 is scaled by 0.119; B) R space and k space plots for the 20 wt % Pt/C catalyst, kw=1 is scaled by 7.403, kw=2 is unscaled and kw=3 is scaled by 0.106.

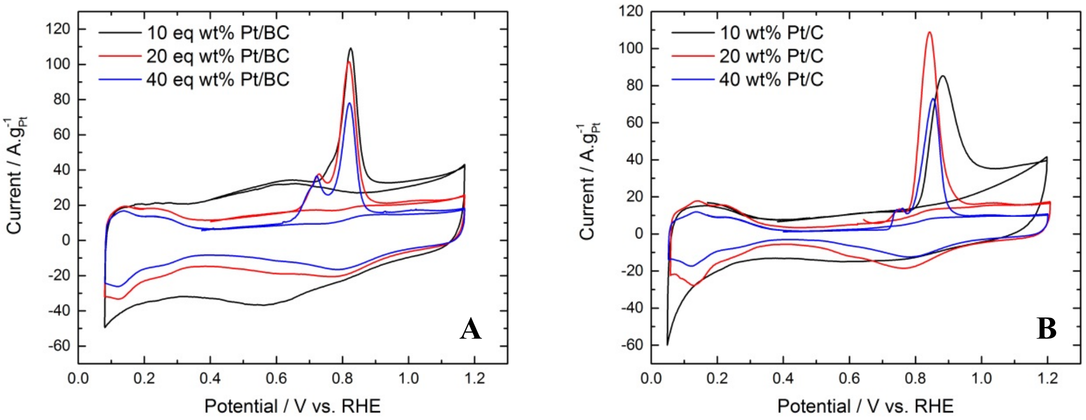

Supplementary Figure 5. CO stripping voltammograms for A) 10 eq wt% Pt/BC, 20 eq wt% Pt/BC and 40 eq wt% Pt/BC catalysts and B) 10 wt% Pt/C, 20 wt% Pt/C and 40 wt% Pt/C catalysts: all experiments performed in 0.1 M $HClO_4$ at room temperature, recorded at 20 mV/s between 0.05 V and 1.2 V vs RHE.

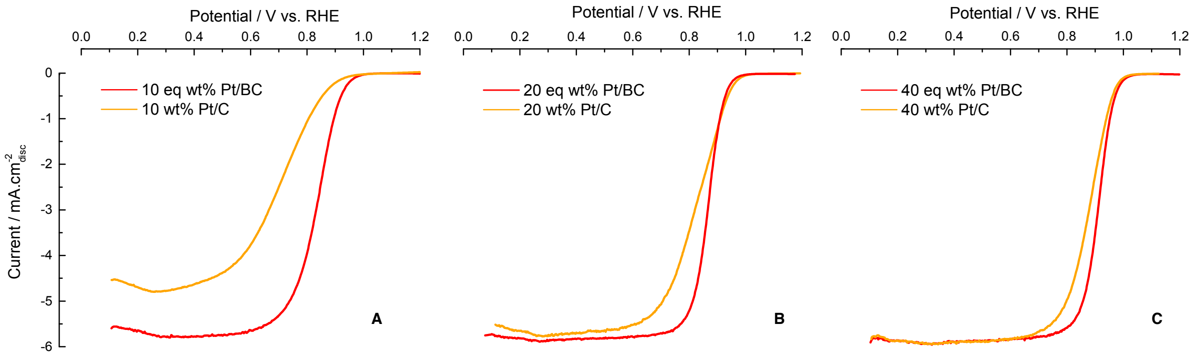

Supplementary Figure 6. Current-Voltage curves as obtained by RDE measurements of A) 10 eq wt% Pt/BC and 10 wt% Pt/C, B) 20 eq wt% Pt/BC and 20 wt% Pt/C, C) 40 eq wt% Pt/BC and 40 wt% Pt/C. The anodic scan was recorded at 1600 rpm between 0.05 V and 1.2 V vs RHE, all experiments performed in 0.1 M $HClO_4$ at room temperature recorded at 20 mV/s using a 5 mm disc; corrected for iR and capacitive charging.

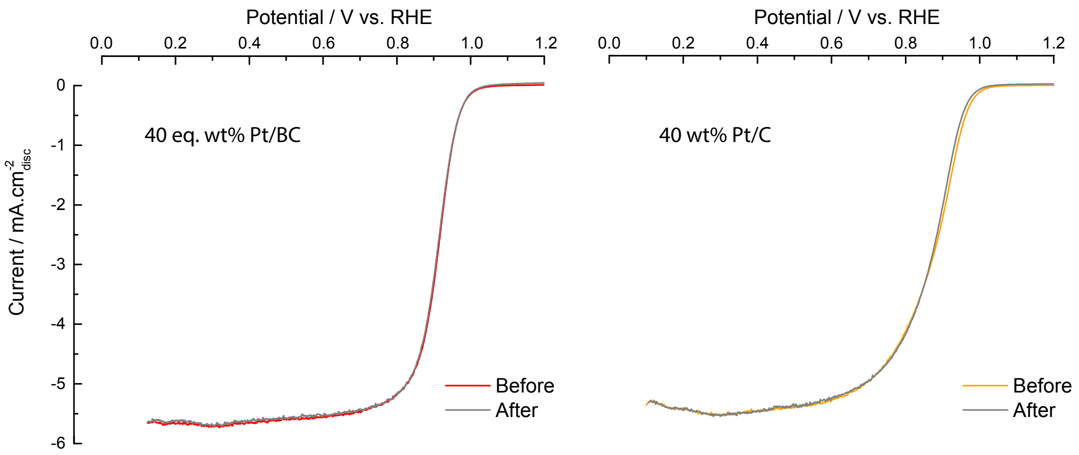

Supplementary Figure 7. Current-Voltage curves as obtained by RDE measurements of the 40 eq. wt% Pt/BC and 40 wt% Pt/C catalysts before and after cycling; the anodic scan was recorded at 1600 rpm between 0.05 V and 1.2 V vs RHE, all experiments performed in 0.1 M $HClO_4$ at room temperature recorded at 20 mV/s; corrected for iR and capacitive charging.

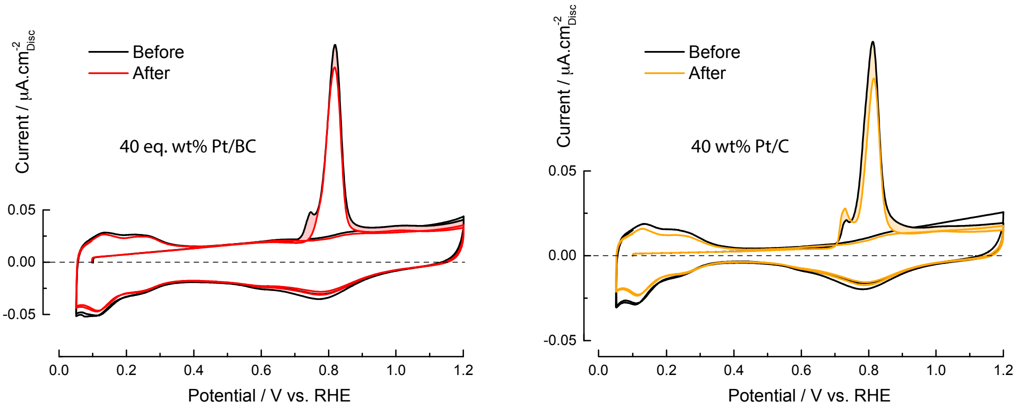

Supplementary Figure 8. CO stripping voltammograms for 40 eq wt% Pt/BC and 40 wt% Pt/C catalysts before and after cycling: all experiments performed in 0.1 M $HClO_4$ at room temperature, recorded at 20 mV/s between 0.05 V and 1.2 V vs RHE.

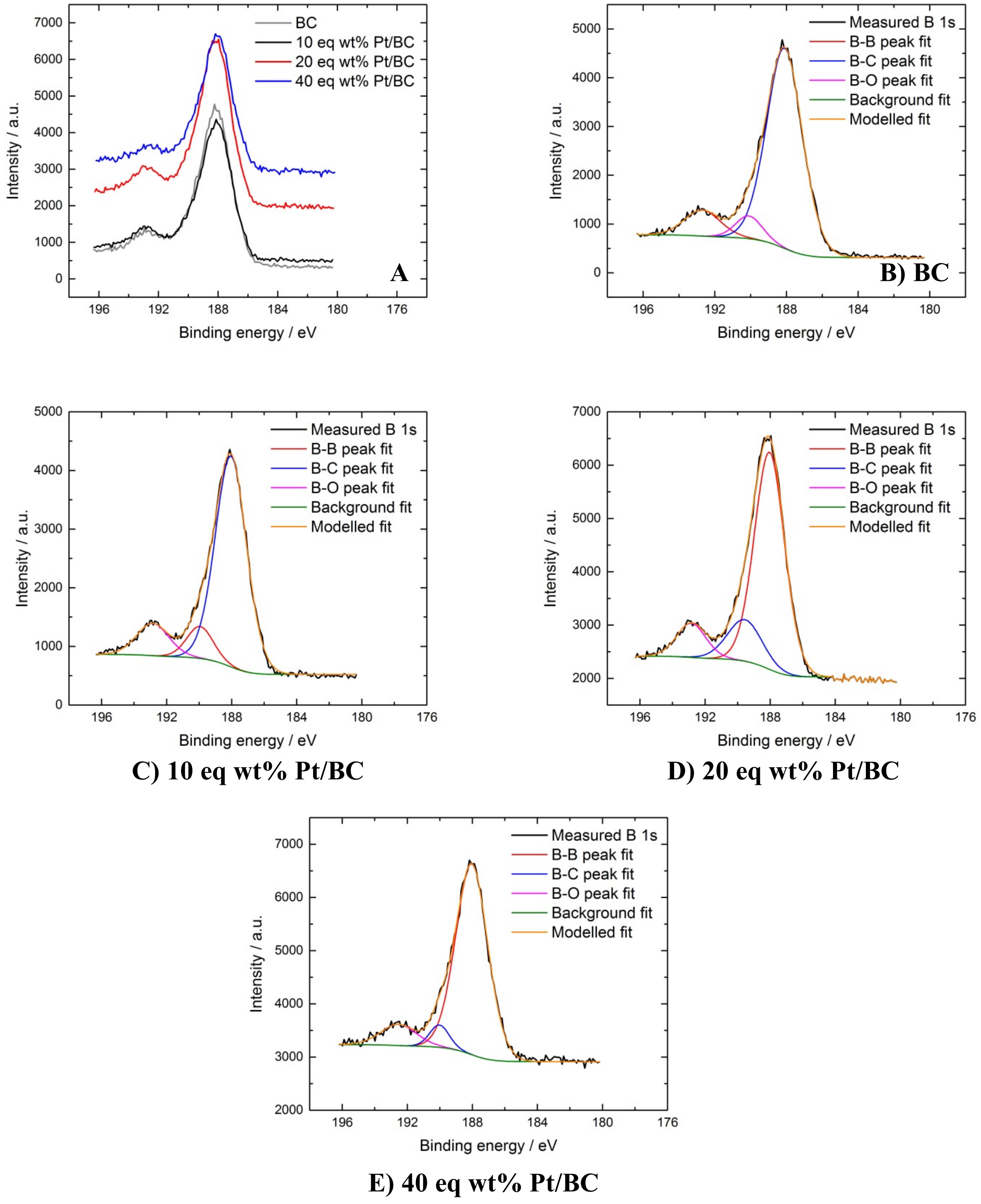

Supplementary Figure 9. XPS spectra of the A) B 1s region of BC and Pt/BC catalysts, B) modelled and actual B1s region of BC support material, C) modelled and actual B1s region of 10 eq wt% Pt/BC, D) modelled and actual B1s region of 20 eq wt% Pt/BC, E) modelled and actual B1s region of 40 eq wt% Pt/BC

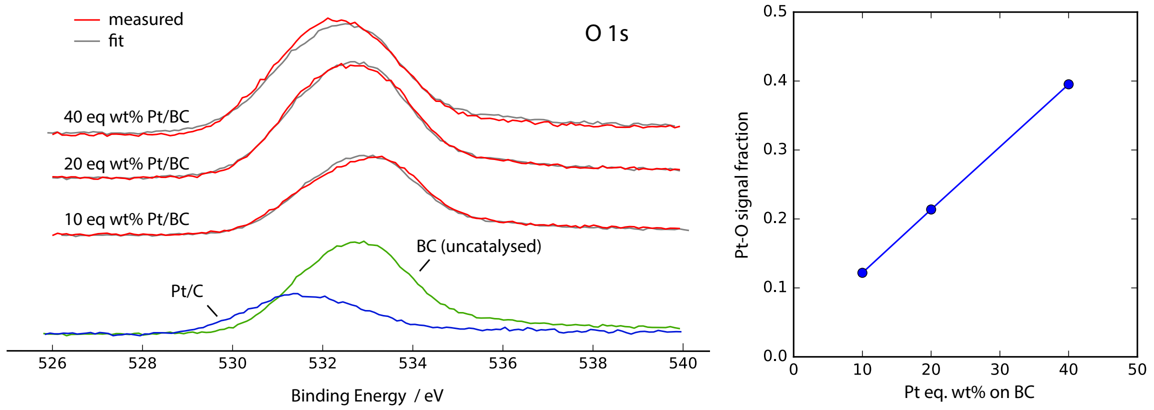

Supplementary Figure 10. XPS spectra of the O 1s region of uncatalyzed BC (green), 20 wt% Pt/C (blue), and 10 eq. wt%, 20 eq. wt%, and 40 eq. wt% Pt/BC catalysts (red); the O 1s region of the BC supported catalysts can be understood as a linear combination of contributions from impurity phase B_2O and oxygen species on Pt (grey); the relative contribution from Pt-O species increases linearly with loading.

Supplementary Table 1: Nominal, actual, and target catalyst loadings on C and BC supports

Nominal wt% Pt/C	Actual wt% Pt/C	Target wt% Pt/BC	Actual wt% Pt/BC
10	9.19	3.05	2.76
20	17.5	6.19	6.86
40	37.85	15.93	12.74

Supplementary Table 2. C 1s chemical shift calculated from DFT

Supercell	# atoms	k-point mesh	Chemical shift Δ [eV]
1x1x1	15	6x6x6	2.18
2x2x2	120	3x3x3	2.28
3x3x3	405	Γ only	2.32

Supplementary Table 3. Literature ORR activity of comparable commercial Pt/C catalysts; temperature correction from Ref. [15] with 10 kJ/mol activation energy; a limiting current j_{lim} of 6 mA/cm$^2_{\mathrm{geo}}$ was used to compensate for O_2 mass transport corrections applied in the literature according to $1/j_s = 1/j_k + 1/j_{\mathrm{lim}}$; data used for comparison in Figure 6 is highlighted; in 0.1 M $HClO_4$ recorded at 20 mV/s and 1600 rpm.

Catalyst	L_{Pt} [μg/cm$^2_{\mathrm{geo}}$]	ECSA [m^2/g$_{\mathrm{Pt}}$]	j_k(30 °C) [μA/cm$^2_{\mathrm{Pt}}$]	j_k(25 °C) [μA/cm$^2_{\mathrm{Pt}}$]	j_s(25 °C) [μA/cm$^2_{\mathrm{Pt}}$]	Comment
**40% Pt/VC (JM)**	**20**	**49**	**538**	**503**	**268**	**Rotational drying; Ref. [1]**
**40% Pt/VC (JM)**	**20**	**49**	**284**	**266**	**182**	**Stationary drying; Ref. [1]**
**19.7% Pt/VC (E-TEK)**	**22**	**61**	**511**	**478**	**223**	**Rotational drying; Ref. [1]**
**19.7% Pt/VC (E-TEK)**	**22**	**55**	**333**	**312**	**186**	**Stationary drying; Ref. [1]**
**20% Pt/Vulcan**	**20**	**61**	**347**	**325**	**190**	**Good film; Ref. [2]**
**20% Pt/Vulcan**	**14.3**	**66**	**305**	**285**	**193**	**Good film; Ref. [2]**
20% Pt/Vulcan	20	57	272	254	168	Intermediate film; Ref. [2]
20% Pt/Vulcan	20	42	230	215	163	Bad film; Ref. [2]

Supplementary References

[1] Garsany, Y., Singer, I. & Swider-Lyons, K. Impact of film drying procedures on RDE characterization of Pt/VC electrocatalysts. *Journal of Electroanalytical Chemistry* 662, 396-406 (2011).

[2] Garsany, Y., Baturina, O., Swider-Lyons, K. & Kocha, S. Experimental Methods for Quantifying the Activity of Platinum Electrocatalysts for the Oxygen Reduction Reaction. *Analytical Chemistry* 82, 6321-6328 (2010).
